# Supplementary material for: Community perspectives of heat and weather warnings for pregnant and postpartum women in Kilifi, Kenya
Source: PLoS One. 2024 Nov 19;19(11):e0313781. doi: 10.1371/journal.pone.0313781 (PMC11575763; doi:10.1371/journal.pone.0313781)
Supplement: Attachment — Submitted filename: PLOS ETHICS LETTER.pdf [file pone.0313781.s001.pdf]

Dr Adelaide Lusambili

Environmental Center, Africa International University,

Email:adelaide.Lusambili@AIU.ac.KE

O5/May/2024

Editorial Manager

PLOS ONE

Dear Edwin Ularte;

I refer to your email as below

" ***Before we can continue with your submission, please upload a copy of the original ethics approval letter, as required by question 1 of the human subject research checklist. If the original letter is not in English, please also provide an English-translated version in the supporting file. Please note that if this documentation is not included when your manuscript is resubmitted, it may be rejected.(Please submit ethics approval of ref numbers: 2020/IERC-94 (v2), 22685, and DOM/KLF/RESCH/vol.1/66.)"***

I respond as follows:

1. The certificate we receive from the National Ethics Approval Committee is attached directly to an email; we do not receive a separate letter. All details pertaining to the study are included in this ethics certificate.

2. Additionally, I have attached other approval documents for the same study, as we usually go through approval processes before the actual data collection:

    - Institutional Ethics Committee from the Aga Khan University approval
    - London School of Hygiene Tropical Medicine Ethics Committee approval
    - County Office Research Ethics Committee approval

Sincerely,

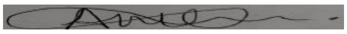

Dr Adelaide Lusambili
