# Supplementary material for: Community perspectives of heat and weather warnings for pregnant and postpartum women in Kilifi, Kenya
Source: PLoS One. 2024 Nov 19;19(11):e0313781. doi: 10.1371/journal.pone.0313781 (PMC11575763; doi:10.1371/journal.pone.0313781)
Supplement: Attachment — Submitted filename: Review comments.docx [file pone.0313781.s002.docx]

**Review comments**

**General comment**

I commend the Authors for conducting the study and the well written manuscript. The study and its findings are of relevance to the community and its inhabitants. Bearing in mind the number of interviews and FGDs that were conducted, perhaps I expected mores quotes in the manuscript.

The dates Internet articles were assessed were not included in the reference section. Also, PLoS ONE makes use of square brackets for reference numbers.

The Authors should also cross-check the length and sub-headings of the abstract section to ensure that it conforms to the guidelines of the Journal.

I recommend that the manuscript be accepted for publication.
