# Supplementary material for: Community perspectives of heat and weather warnings for pregnant and postpartum women in Kilifi, Kenya
Source: PLoS One. 2024 Nov 19;19(11):e0313781. doi: 10.1371/journal.pone.0313781 (PMC11575763; doi:10.1371/journal.pone.0313781)
Supplement: Attachment — Submitted filename: Review_Kilifi_HeatWarnings.docx [file pone.0313781.s003.docx]

**Review: A Qualitative Study of Heat Early Weather Warnings for Pregnant Women and Children in Kilifi, Kenya**

Abstract – overall this is well-written, but please ensure it’s within the journal’s word limit

Introduction –

- Please include what the UN early warning for all is – what’s their aim more specifically?

Methods –

- Subheading “Interviewing” should be “Interviews”
- Remove period after ‘criteria’ subheading

Results –

- Remove “scorching” from your narrative in the sentence before it also appears in the IDI quote

Discussion –

- Check consistency of terminology compared to earlier in the paper “early warning weather” vs “early weather warning”
- It seems worth stating that more research is needed to ascertain whether traditional weather indications stay true in the changing climate (ie. Frogs croaking <2 weeks before rains). Even when weather patterns shift, it’s likely (?) that indicators shift with them
