# Supplementary material for: Community perspectives of heat and weather warnings for pregnant and postpartum women in Kilifi, Kenya
Source: PLoS One. 2024 Nov 19;19(11):e0313781. doi: 10.1371/journal.pone.0313781 (PMC11575763; doi:10.1371/journal.pone.0313781)
Supplement: Attachment — Submitted filename: RESPONSE TO THE EDITOR.docx [file pone.0313781.s004.docx]

**RESPONSE TO THE EDITOR**

Dear Dr. Lusambili,

We've checked your submission, and before we can proceed, we need you to address the following issues: In the online submission form, you indicate that your data is not available for proprietary reasons and provide a contact point for accessing this data. Please note that your current contact point is a co-author on this manuscript. According to our Data Policy, the contact point must not be an author on the manuscript and must be an institutional contact, ideally not an individual. Please revise your data statement to a non-author institutional point of contact, such as a data access or ethics committee, and send this to us via return email. Please also include contact information for the third party organization, and please include the full citation of where the data can be found. We've returned your manuscript to your account. Please resolve these issues and resubmit your manuscript within 21 days. If you need more time, please email the journal office at [plosone@plos.org](mailto:plosone@plos.org). We are happy to grant extensions of up to one month past this due date. If we do not hear from you within 21 days, we will withdraw your manuscript.

**OUR RESPONSE**

Our study is qualitative, involving a small group of participants from rural areas. Due to the sensitive nature of the data and our commitment to maintaining confidentiality, releasing the data could potentially identify individuals. As a result, data access will only be available upon reasonable request through the Aga Khan University Research Ethics Office.

For inquiries, please contact:
Aga Khan University Research Ethics Office
Email: research.office@aku.edu. Since Aga Khan University in Kenya conducted the study, there is no third-party contact. We hope this approach meets your requirements and are happy to provide further information if needed. The revised document has been resubmitted.
