# Supplementary material for: Community perspectives of heat and weather warnings for pregnant and postpartum women in Kilifi, Kenya
Source: PLoS One. 2024 Nov 19;19(11):e0313781. doi: 10.1371/journal.pone.0313781 (PMC11575763; doi:10.1371/journal.pone.0313781)
Supplement: Attachment — Submitted filename: Review comments.docx [file pone.0313781.s005.docx]

**Review comments**

**General comments**

Once again, I commend the Authors for the well written manuscript as submitted. Keep up the good work.

I recommend that the manuscript should be accepted for publication.

**Observations.**

1. Even though acronyms were introduced when they were used for the first time in the abstract section, the case was not the same in the body of the manuscript. Authors should refer to lines 149 and 262.
2. Tables 1 and 2 should be titled ‘Participants’ profile’ instead of ‘Socio-demographics’.
